# Editorial: Rising stars in: quantitative psychology and measurement 2021

**DOI:** 10.3389/fpsyg.2024.1444644

**Published:** 2024-08-07

**Authors:** Pietro Cipresso, Alessandro Giuliani

**Affiliations:** ^1^Department of Psychology, University of Turin, Turin, Italy; ^2^Environment and Health Department, Istituto Superiore di Sanità (National Institute of Health), Rome, Lazio, Italy

**Keywords:** psychometrics, quantitative psychology, mathematical psychology, modeling, rising star

The “*Rising stars in: quantitative psychology and measurement 2021*” Research Topic, presented in Frontiers in Psychology, is a compelling Research Topic that highlights the innovative work of internationally recognized researchers in the early stages of their independent careers. This Research Topic not only showcases the diversity of research across the breadth of Quantitative Psychology and Measurement but also marks significant advances in theory, experiment, and methodology, with direct applications to challenging and relevant problems in the field.

The focus of this Research Topic is diverse, including but not limited to:

1. Interdisciplinary efforts utilizing statistical mechanics-inspired methods for identifying phase transition points in psychotherapy processes.
2. The application of complex network formalism for defining symptoms constellations and pathogenetic mechanisms.
3. Innovative uses of multidimensional statistics for analyzing questionnaires.
4. Data mining of unstructured data.

These themes underscore a commitment to exploring new frontiers in psychological research, emphasizing the development of novel methods and applications. The articles in this Research Topic have been carefully selected by the editors, recognizing each contributing researcher's potential to significantly influence future directions in their respective fields.

The editors of this topic, Pietro Cipresso from the University of Turin and Alessandro Giuliani from the National Institute of Health, have curated a series of articles that exemplify the progress made in Quantitative Psychology and Measurement over the past decade. They have also set an agenda for addressing future challenges, offering a comprehensive overview of the state of the art in this research area.

This Research Topic is not just a testament to the achievements in this field but also a guidepost for future research directions, inspiring and directing researchers with its forward-looking perspective. Each article represents a unique contribution to the field, showcasing the depth and range of research being conducted by rising stars in Quantitative Psychology and Measurement.

This year's focus is highlighted by 8 pioneering studies.

The article “*Development of a computerized adaptive test for problematic mobile phone use*” underscores the technological integration in psychological assessment (Liu et al.). This research demonstrates a significant leap in measurement precision and efficiency, setting a precedent for future investigations. The articles in this Research Topic collectively represent a paradigm shift in quantitative psychological methods, embracing technology to address contemporary issues with a level of sophistication and accuracy hitherto unseen. Each study contributes uniquely to our understanding of human behavior in an increasingly digital world, reflecting the cutting-edge of psychological research.

*Don't worry about the anchor-item setting in longitudinal learning diagnostic assessments* is a study addresses the relevance and impact of anchor-item settings in longitudinal learning diagnostic assessments (LDAs) (Yu et al.). Through simulation studies, it investigates whether setting specific items as anchors affects classification accuracy in these assessments. The findings suggest that the inclusion or exclusion of anchor items, as well as their specific configuration, does not significantly impact classification accuracy. This research contributes to the practical application of LDAs by providing insights into the effectiveness of different anchor-item settings.

The study titled *Evidence of validity of internal structure of the FACIT-Sp-12 in Brazilian adolescents with chronic health conditions* validates the FACIT-Sp-12 scale for assessing spiritual wellbeing in Brazilian adolescents with chronic illnesses (Alvarenga et al.). It employs Exploratory Factor Analysis (EFA), Confirmatory Factor Analysis (CFA), and Item Response Theory (IRT) to examine the scale's reliability and structural validity. The research reveals a one-dimensional scale structure, offering insights into its application for this demographic.

The article *Outlining a novel psychometric model of mental flexibility and affect dynamics* delves into the concept of mental flexibility and its relationship with affect dynamics (Borghesi et al.). It proposes a new psychometric model to understand this relationship using Markovian chain analysis. The study contributes to the field by offering a novel and comprehensive understanding of the interplay between mental flexibility and affect dynamics, providing a fresh perspective on these complex psychological processes.

The article *Psychological assessment in infertility: a systematic review and meta-analysis* provides a critical evaluation of the psychological impact of infertility (Tavousi et al.). It delves into various psychological assessment tools used in this context, analyzing their effectiveness and the nuances in measuring the psychological aspects related to infertility. This study offers a detailed synthesis of existing research, presenting a comprehensive view of the psychological dimensions and challenges faced by individuals experiencing infertility. It also identifies gaps in the current research, suggesting areas for future investigation to enhance the understanding and support for those affected by infertility.

The article *Psychometric properties of the Chinese revision of The Pitt Wellness scale for people in the university environment* investigates the adaptation and validation of the Pitt Wellness Scale in a Chinese university context (Yan et al.). This study is significant for its cultural adaptation of a wellbeing assessment tool, addressing the unique aspects of the Chinese university environment. The results demonstrate the scale's satisfactory psychometric properties, highlighting its potential as a reliable instrument for assessing wellbeing in this specific demographic. This adaptation offers a valuable contribution to the field of quantitative psychology and measurement, particularly in the context of cross-cultural research and application.

The article *Psychometric review of the perceived stress scale under CFA and Rasch models in Lima, Peru* focuses on the validation of the Perceived Stress Scale (PSS) in the Peruvian context. Employing Confirmatory Factor Analysis (CFA) and Rasch models, the study evaluates the scale's factorial structure, confirming its adaptability and reliability (Boluarte-Carbajal et al.). The research provides important insights into the scale's applicability in different cultural contexts, contributing significantly to the field of stress measurement and psychological assessment.

The article *What matters when examining the performance of salespersons? Analyzing the boundary conditions of personal dispositional factor* focuses on the impact of performance appraisal (PA) on job meaningfulness (JM) in salespersons (Naeem et al.). It evaluates the roles of developmental and evaluative PA and explores how external locus of control (ELOC) moderates these relationships. The study reveals that developmental PA significantly influences JM, whereas evaluative PA does not. Additionally, ELOC moderates the relationship between developmental PA and JM, offering insights into personal factors affecting workplace outcomes.

## Author contributions

PC: Conceptualization, Writing – original draft, Writing – review & editing. AG: Conceptualization, Writing – review & editing.

